# Suppression of KRas-mutant cancer through the combined inhibition of KRAS with PLK1 and ROCK

**DOI:** 10.1038/ncomms11363

**Published:** 2016-05-19

**Authors:** Jieqiong Wang, Kewen Hu, Jiawei Guo, Feixiong Cheng, Jing Lv, Wenhao Jiang, Weiqiang Lu, Jinsong Liu, Xiufeng Pang, Mingyao Liu

**Affiliations:** 1 Shanghai Key Laboratory of Regulatory Biology, Institute of Biomedical Sciences and School of Life Sciences, East China Normal University, Shanghai 200241, China; 2 Cancer Institute, Fudan University Shanghai Cancer Center, Department of Oncology, Shanghai Medical College, Fudan University, Shanghai 200032, China; 3 State Key Laboratory of Biotherapy/Collaborative Innovation Center for Biotherapy, West China Hospital, West China Medical School, Sichuan University, Chengdu, Sichuan 610041, China; 4 Bioinformatics and Systems Medicine Laboratory, Department of Biomedical Informatics, Vanderbilt University Medical Center, Nashville, Tennessee 37203, USA; 5 Department of Pathology, The University of Texas MD Anderson Cancer Center, Houston, Texas 77030, USA; 6 Institute of Biosciences and Technology, Department of Molecular and Cellular Medicine, Texas A&M University Health Science Center, Houston, Texas 77030, USA

## Abstract

No effective targeted therapies exist for cancers with somatic *KRAS* mutations. Here we develop a synthetic lethal chemical screen in isogenic *KRAS*-mutant and wild-type cells to identify clinical drug pairs. Our results show that dual inhibition of polo-like kinase 1 and RhoA/Rho kinase (ROCK) leads to the synergistic effects in *KRAS*-mutant cancers. Microarray analysis reveals that this combinatory inhibition significantly increases transcription and activity of cyclin-dependent kinase inhibitor p21^WAF1/CIP1^, leading to specific G2/M phase blockade in *KRAS*-mutant cells. Overexpression of p21^WAF1/CIP1^, either by cDNA transfection or clinical drugs, preferentially impairs the growth of *KRAS*-mutant cells, suggesting a druggable synthetic lethal interaction between KRAS and p21^WAF1/CIP1^. Co-administration of BI-2536 and fasudil either in the LSL-KRAS^G12D^ mouse model or in a patient tumour explant mouse model of *KRAS*-mutant lung cancer suppresses tumour growth and significantly prolongs mouse survival, suggesting a strong synergy *in vivo* and a potential avenue for therapeutic treatment of *KRAS*-mutant cancers.

## Abstract (web summary)

KRas is frequently mutated in multiple cancer types; identifying drugs to treat such cancers is a good therapeutic strategy. Here, the authors perform a synthetic lethal screen in mice and show that inhibiting Plk1 and ROCK results in the inhibition of tumour growth by increasing expression of the tumour suppressor p21.

## Graphical abstract

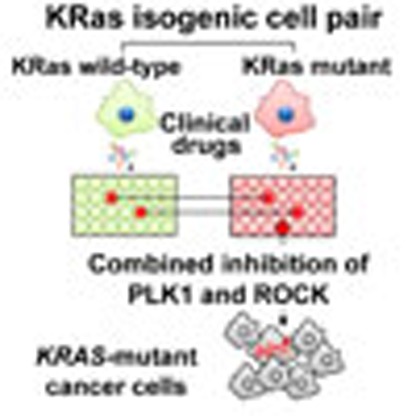

Oncogenic mutations in the GTPase protein RAS (NRAS, KRAS and HRAS) occur in up to a third of all human cancers^1^,^2^,^3^. KRAS is the principal isoform of RAS, and somatic mutations in *KRAS* are associated with over 21% of all cancers; a particularly high frequency of *KRAS* mutations is observed in pancreatic, colorectal and lung cancers, three of the top four leading causes of new cancer deaths^4^. *KRAS*-mutant tumours exhibit oncogene addiction^5^,^6^, and KRAS seems to be an ideal target for cancer therapeutics. However, 30 years after its characterization, mutant KRAS still poses a significant therapeutic challenge and is associated with poor prognosis and resistance to existing therapies^7^,^8^. Alberto and colleagues recently drew an analogy between the challenges of tackling mutant KRAS and climbing Mount Everest^8^. The lack of suitable small-molecule binding sites in its protein structure makes RAS difficult to target^1^. Partly due to this issue, direct pharmacologic targeting of activated RAS protein with small molecules seems to be an impossible task, although small molecules that bind to allosteric regulatory sites on KRAS (G12C) have shown preclinical benefits^9^. To overcome this limitation, investigators have explored other potential therapeutic approaches, such as indirectly targeting mutant KRAS, developing drugs to inhibit downstream KRAS effectors or using unbiased searches for targets that are synthetically lethal with mutant *KRAS*^3^,^10^.

The majority of the efforts over the past few years have aimed at developing drugs that target KRAS downstream effectors. However, targeting a single KRAS effector or a combination of multiple downstream effectors showed modest or no clinical responses against mutant KRAS cancers in early-phase clinical trials^8^,^11^,^12^,^13^; this is probably because KRAS activates multiple parallel networks and is subject to uncontrolled signalling feedback. Recent studies have taken advantage of *KRAS* oncogene addiction to search for synthetic lethal partners of mutant KRAS. Several genome-wide RNA interference (RNAi) screens and other technologies have identified a list of candidate genes, including TANK-binding kinase 1 (*TBK1*)^14^, serine-threonine kinase *STK33* (ref. 15) and heat-shock protein 90 (*HSP90*; the inhibition of HSP90 involves the degradation of STK33)^16^, polo-like kinase 1 (*PLK1*)^17^,^18^, cyclin-dependent kinase 4 (*CDK4*)^19^, transcription factor (TF) *GATA2* (refs 20, 21), evolutionarily conserved gene enhancer of rudimentary homologue (*ERH*)^18^, transforming growth factor β-activated kinase 1 (*TAK1*)^22^, anti-apoptotic BH3 family member *BCL-XL*^23^, mitotic regulators^17^,^21^, proteasome and topoisomerase components^17^,^20^,^21^ and genes that are involved in glucose metabolism (for example, hexokinase 2)^24^. Although only a modest overlap was found among these identified candidates (probably due to differences in cell types and experimental setting) and some candidate genes lack effective inhibitors (for example, the TF GATA2), the strategy aimed at synthetic lethality did open up a new avenue to treat *KRAS*-driven cancers. To advance the discovery of drugs against mutant KRAS tumours, synthetic lethal chemical screens have emerged and represent a direct and complementary approach to identifying drugs that target the essential signalling networks for the growth of *KRAS*-mutant tumours. More importantly, such an approach could also provide immediately usable translational therapeutic strategies by identifying drugs that are already available in clinic.

To achieve more potent efficacy and less toxicity, a ‘cocktail' of drugs is now often given to avoid primary or acquired resistance to single agents after a period of administration^25^, especially in *KRAS*-mutant cancers^23^,^26^. The combined inhibition of KRAS downstream effectors has shown toxicity and disappointing efficacy in clinical testing^27^, therefore searching for novel and clinically applicable anti-RAS drug pairs is of great importance.

In this study, we employ synthetic lethal drug screening plus a combinatorial strategy using a panel of clinical agents. We find that drugs targeting KRAS synthetic lethal genes account for a large proportion of observed synergies and identify several novel and unique genotype-selective synergistic drug pairs within the panel. We further confirm the selective anti-RAS efficacy of combined inhibition of PLK1 and Rho signalling *in vitro* and *in vivo*. This combination increases the expression of p21^WAF1/CIP1^ in *KRAS*-mutant cells in a p53-independent manner. Notably, the overexpression of p21^WAF1/CIP1^ selectively kills cells harbouring *KRAS* mutations while sparing wild-type cells. Our findings suggest a drug combination that disrupts KRAS synthetic lethal dependency, which could be therapeutically exploited to inhibit *KRAS*-driven cancers.

## Results

### A synthetic lethal drug screen

To identify innovative drug combinations to treat *KRAS*-mutant cancers, we performed a synthetic lethal chemical screen. We evaluated the impact of the *KRAS*-mutant genotype on a panel of agents that directly or indirectly targeted RAS effector pathways and therefore had a potential for synthetic lethal interaction with mutant KRAS (Supplementary Table 1). Because we aimed to employ synthetic lethal drug screening plus a combinatorial strategy, we assume that the drugs targeting pathways upstream of RAS, downstream of RAS or mutant RAS-dependent pathways may coordinate with each other to produce more profound actions to kill *KRAS*-mutant tumours by unrivalled mechanisms or signalling crosstalk.

We performed the primary screening in immortalized human ovarian surface epithelial cells (T29) and their transformed isogenic counterparts that expressed mutationally activated KRAS^V12^ (designated T29Kt1)^28^. The concentrations required to inhibit 50% of cell growth (half-maximal inhibitory concentration, IC_50_) were calculated (Supplementary Table 1). Most inhibitors showed selectivity for the *KRAS*-mutant genotype as a single agent (for example, BI-2536), but several agents elicited incomplete growth inhibition. The combinatorial screening of these drugs was then performed to achieve full growth inhibition and increase the differential sensitivity between *KRAS*-mutant and wild-type cells. When tested, T29Kt1 cells were not preferentially sensitive to gefitinib, a small-molecule inhibitor of the epidermal growth factor receptor (EGFR), as the IC_50_'s in T29Kt1 and T29 cells were rather comparable. These results were consistent with the reports that tumours bearing activating mutations within *KRAS* are highly negative or resistant to anti-EGFR therapy^29^,^30^.

**Figure 1:**
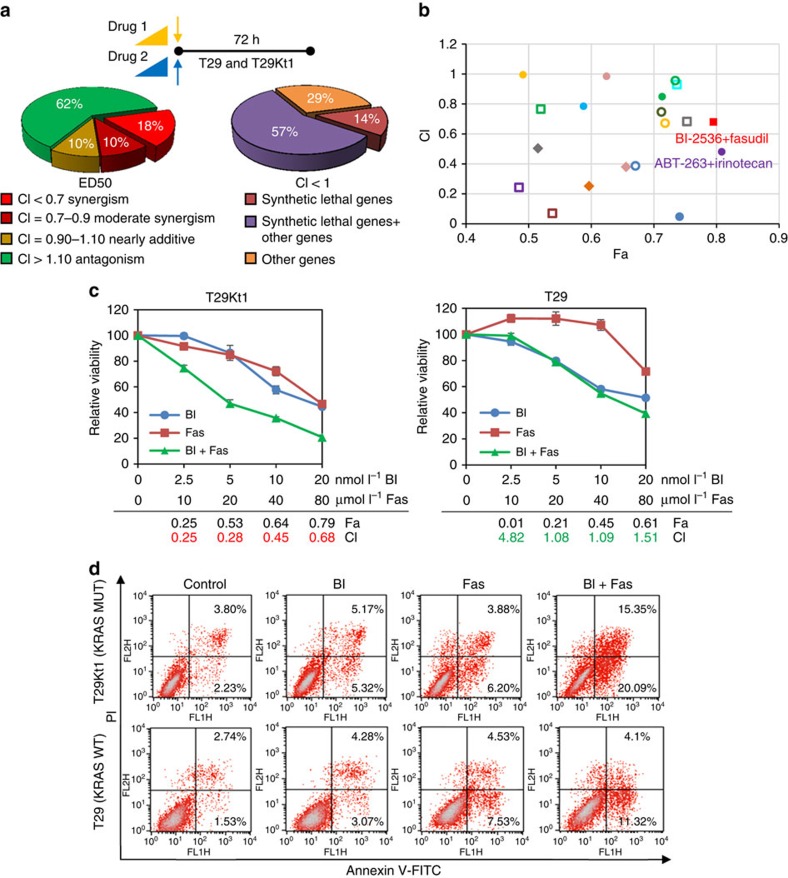
A synthetic lethal chemical screen reveals that *KRAS*-mutant cells are selectively sensitive to the combined inhibition of PLK1 and ROCK. (**a**) Schematic of the combinations tested and the drug interaction signatures. Twenty clinical drugs were tested in pairwise combinations (a total of 190 pairs) in T29Kt1 cells harbouring a *KRAS* mutation. Drugs were added at a relevant fixed ratios (IC_50_ ratios, see also Supplementary Table 1) at four concentration combinations in each representative drug pair. The cell viability was determined. Left: compilation of the total number of drug pair synergies, moderate synergies, nearly additive interactions and antagonistic interactions. The combination index (CI) was calculated using CalcuSyn software (Version 2; Biosoft) as described in the Methods section. Right: the frequencies at which the drug target gene types appear in the synergy cluster (CI<1). The oncogenic *KRAS* synthetic lethal genes accounted for the largest proportion of synergies specific to *KRAS*-mutant cells. (**b**) The Fa–CI plot. The Fa (fraction affected by the dose) and CI value of two drugs at their combination of IC_50_'s were listed in *X* and *Y* axes and synergistic pairs with CI<1 were shown. The combination of BI-2536 and fasudil exhibited leading therapeutic efficacy and applicable potential. (**c**) The cytotoxicity of BI-2536 and fasudil. T29Kt1 and T29 cells were incubated with increasing concentrations of BI-2536 (BI) and fasudil (Fas) alone or in combination for 72 h, and the cell viability was determined. The CI and Fa values for the combination of BI-2536 and fasudil were calculated. The averages and error bars represent the mean±s.d. from three independent experiments. (**d**) Percentage of apoptotic cells was determined by Annexin-V and propidium iodide staining after BI-2536 (10 nmol l^−1^) and fasudil (40 μmol l^−1^) treatment alone or in combination for 72 h in T29Kt1 and T29 cells.

To explore the most effective drug combinations, we generated a mixture of two drugs at their equipotent ratio (at the ratio of their IC_50_'s) and treated T29Kt1 cells with serial dilutions of the mixture (1:1, 1:2, 1:4 and 1:8), resulting in four pairwise combinations per drug pair in parallel with single-agent controls. The combination index (CI) was calculated using CalcuSyn software (Version 2; Biosoft) to analyse the interaction (synergistic, additive or antagonistic) (Fig. 1a). The combinations were often antagonistic (62%) in T29Kt1 cells, while only 28% (synergism 18% and moderate synergism 10%) of the drug pairs showed a pattern of extensive synergies (Fig. 1a, left). To identify genotype differences in the synergistic drug pairs (CI<1), we categorized the inhibitors as either targeting synthetic lethal genes or other genes. Inhibitors of synthetic lethal genes contributed to over 70% of the synergies observed (Fig. 1a, right), indicating that synthetic lethal genes play an indispensable role in the growth of *KRAS*-mutant cancers and represent applicable targets for treatment. We analysed the Fa (fraction affected by the dose) and CI value of each pair of two drugs at their IC_50s_, and found that the drug pair of BI-2536 and fasudil was in the leading rank and achieved satisfying Fa value in Fa–CI plot (Fig. 1b). BI-2536, a selective inhibitor of PLK1, effectively inhibits different types of tumour in phase II clinical trials. In addition, *PLK1* gene was identified as a synthetic lethal partner of RAS oncogene^17^. Fasudil, a RhoA/Rho kinase (ROCK) inhibitor, has been approved in Japan and China for treatment of cerebral vasospasm, stroke and hypertension. Although the Fa values of drug pairs of ABT-263 (Bcl-xL inhibitor) with irinotecan (topoisomerase inhibitor) was higher than that of BI-2536/fasudil, we collectively considered their drug target, clinical safety and progression, and finally chose the drug pair of BI-2536 and fasudil. Next, we extensively investigated the effects of combined inhibition of the PLK1 and Rho signalling pathways on *KRA*S-mutant cancers *in vitro* and *in vivo*.

Compared with BI-2536 or fasudil alone, BI-2536 plus fasudil exhibited enhanced cytotoxicity in T29Kt1 cells. The CI values were all <0.7, indicating a strongly synergistic interaction between BI-2536 and fasudil in *KRAS*-mutant cancer cells. In contrast, this combination was antagonistic in *KRAS* wild-type T29 cells (Fig. 1c). This combination consistently led to significantly more apoptosis than either agent alone (Fig. 1d) in T29Kt1 cells, as observed by an Annexin-V/propidium iodide apoptosis assay. Synergy of combined inhibition of PLK1 and ROCK was also got from another drug pair of BI-6727 and Y-27632 (Supplementary Fig. 1). These results suggest that the combined inhibition of PLK1 and ROCK is a novel regimen for inhibiting *KRAS*-mutant cancer cells.

### The inhibition of PLK1 and ROCK leads to enhanced toxicity

**Figure 2:**
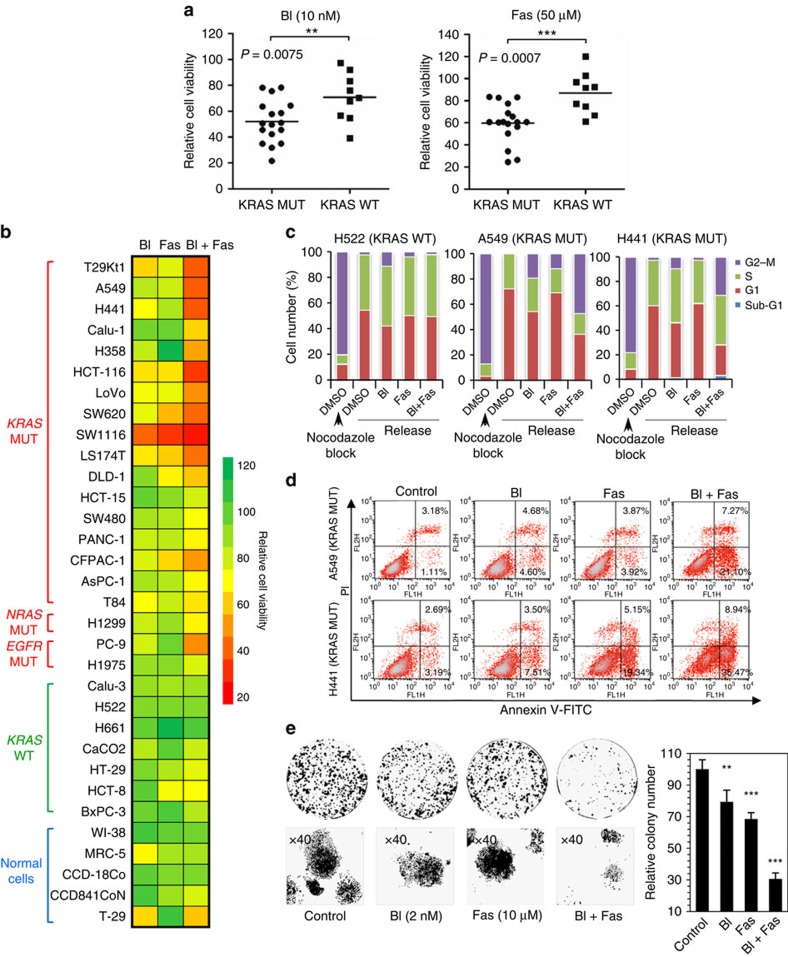
*KRAS*-mutant cancer cells are significantly sensitive to the pharmacologic inhibition of PLK1 and ROCK. (**a**) Seventeen *KRAS*-mutant (KRAS MUT) and nine wild-type (KRAS WT) cancer cell lines were treated with the indicated concentrations of BI-2536 or fasudil for 72 h; the dots represent the cell viability normalized to no drug treatment. The bars indicate the means. Student's *t*-tests were performed between the MUT and WT groups; **P<0.01; ***P<0.001. (**b**) Thirty-two cell lines carrying different *KRAS* genotypes were treated with BI, Fas or the combination of BI and Fas for 72 h. The percentage of viable cells was colour coded in a heatmap. (**c**) H522 (KRAS WT), A549 (KRAS MUT) and H441 (KRAS MUT) cells were treated with BI-2536 (2 nmol l^−1^), fasudil (10 μmol l^−1^) or the combination (BI-2536+fasudil) for 48 h after synchronization and release. The cell cycle distribution was analysed by flow cytometry using propidium iodide staining. (**d**) A549 and H441 (KRAS MUT) cells were treated with BI-2536 (2 nmol l^−1^), fasudil (10 μmol l^−1^) or a combination of BI and Fas for 72 h, and the percentage of apoptotic cells (Annexin positive) was determined by Annexin-V and propidium iodide staining. (**e**) A549 cells (1,000) were plated in 60-mm dishes and treated with dimethylsulphoxide, BI-2536 (2 nmol l^−1^), fasudil (10 μmol l^−1^) or a combination of BI and Fas for 7 days. The cell colonies were stained with crystal violet and counted. The relative number of colonies was calculated by normalization to control as 100%. The values represent the mean±s.d. of three independent assays; Student's *t*-tests were performed; **P<0.01; ***P<0.001. MUT, mutant; WT, wild type.

To verify the synergistic effect of the synthetically lethal genes PLK1 and ROCK, we evaluated a panel of cell lines from lung, colon and pancreatic cancers in which a *KRAS* mutation is the predominant oncogenic alteration^3^, as well as four normal human cell lines (fibroblasts/epithelial cells; Supplementary Table 2). Human cancer cell lines bearing *KRAS* mutations tended to be more sensitive to either BI-2536 or fasudil than *KRAS* wild-type ones (Fig. 2a; *P*<0.01 or *P*<0.001, Student's *t*-tests). Intriguingly, the combination of BI-2536 and fasudil at low doses led to a more robust reduction of cell viability than either single agent alone in *KRAS*-mutant, but not wild-type, cancer cells (Fig. 2b; Supplementary Fig. 2a). More importantly, this drug pair had minimal effects on normal human fibroblasts or epithelial cells (Fig. 2b), indicating that the drug pair is directly cytotoxic in cancer cells.

We next analysed the cell cycle distribution of H522 (*KRAS* wild-type), A549 (*KRAS*-mutant) and H441 (*KRAS*-mutant) cells after exposure to BI-2536/fasudil or either single drug alone. Our results showed that BI-2536/fasudil led to a profound G2/M arrest in *KRAS*-mutant cells but only modestly affected the cell cycle profile of wild-type cells (Fig. 2c). Moreover, BI-2536/fasudil induced marked apoptosis in *KRAS*-mutant A549 and H441 cells, with a sevenfold increase in the number of apoptotic cells compared with the untreated controls (Fig. 2d). A long-term clonogenicity assay was then performed to assess whether BI-2536 plus fasudil had the capacity to cause irreversible growth arrest in *KRAS*-mutant cells. We observed a marked decrease in colony number along with colony size in the combination group (Fig. 2e). Taken together, these results suggest that this novel drug pair can rather effectively inhibit the growth of a large proportion of *KRAS*-mutant cancer cells of different tissue origins.

### The p53 pathway is involved in the sensitivity of drug pair

**Figure 3:**
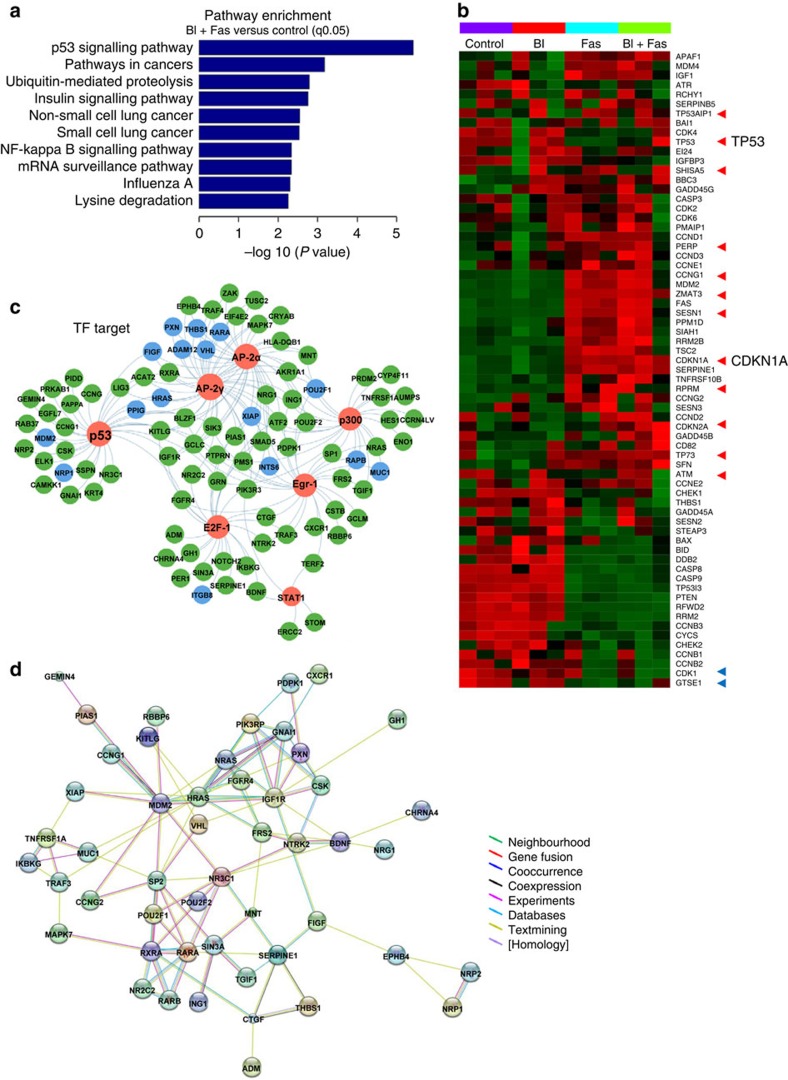
The p53 signalling pathway is involved in the sensitivity of BI-2536/fasudil in *KRAS*-mutant cancers. (**a**) Pathway enrichment of the differentially expressed genes in A549 cells after BI-2536+fasudil treatment. For enrichment analysis, the subsets of upregulated genes were used. The bar plot shows the top 10 enrichment score (−log (*P* value)) value of the significant enrichment pathway. The *P* value cutoff was 0.05 and denotes the significance of the pathway correlated to the conditions from three independent experiments. (**b**) Heatmap coloured according to the *Z* scores of the expression value, showing the expression profile of all of the genes in the p53 pathway. The arrow points to DEGs with FC≥2 and FDR≤0.05. The red arrows represent genes that are upregulated, and the blue arrows represent genes that are downregulated. *TP53* and *CDKN1A*, primary members of this pathway, are indicated. (**c**) Differentially expressed disease genes that are explained by enriched TF. Red node, TFs; blue node, non-small cell lung cancer-related genes; green node, lung cancer-related genes. (**d**) Protein–protein interaction networks, functional modules consisting of the genes downstream of the enriched TF.

To identify the potential mechanisms that underlie the enhanced activity of the combined PLK1 and ROCK inhibition, we conducted a microarray analysis to examine the gene expression profiles in *KRAS*-mutant A549 cells treated with BI-2536/fasudil or vehicle control. The differentially expressed genes (DEGs) were identified based on a false discovery rate threshold of 0.05. Pathway enrichment analysis, an analysis that maps genes to the KEGG pathways, revealed that the p53 signalling pathway had the highest number of significantly upregulated genes (Fig. 3a). The most statistically significant changes in the gene expression of the p53 signalling pathway components were shown in the heatmap (Fig. 3b). Important genes that are involved in the function of the p53 pathway^31^, including CDKs, CDKN1A, ATM and others, were highlighted. Both single agents led to the changes in gene expression in different directions to some extent, but the combination of BI-2536 and fasudil further enhanced these gene expression patterns (Fig. 3b).

Oncogenic signalling networks typically exhibit crosstalk between multiple signalling pathways, rendering single-protein measurements ineffective in predicting complex cellular responses to drugs^32^. To investigate the integrated regulatory mechanism involved in the selectivity of BI-2536/fasudil, we further performed transcription factor-binding site (TFBS) enrichment analysis to predict the underlying TF network based on our DEG profiles. The University of California Santa Cruz genome browser (UCSC) TFBS conserved track data^33^ were used to reduce the false positive rate in our analysis (Supplementary Table 3). TFBS analysis suggests that several TFs, such as p53, E2F and AP2, were activated by BI-2536/fasudil, and that most of the DEGs that are targets of these activated TFs, which is related to disease genes in lung cancer (green) and non-small cell lung cancer (NSCLC; blue) according to the MalaCards database (Fig. 3c). We further analysed the protein–protein interaction networks using data from the STRING database. The results showed that the DEGs formed a densely connected network (Fig. 3d), suggesting that these genes work as a functional module at the protein level.

### The inhibition of PLK1 and ROCK upregulates p21^WAF1/CIP1^

**Figure 4:**
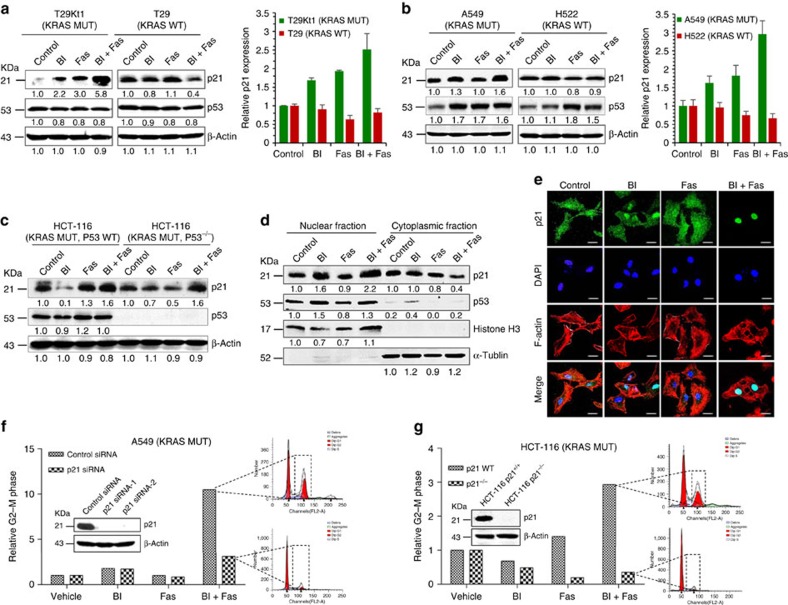
The combined inhibition of PLK1 and ROCK leads to the overexpression of p21^WAF1/CIP1^ in *KRAS*-mutant cells but not wild-type cells. (**a**) The levels of p21 protein and mRNA expression in isogenic T29Kt1/T29 cells. Cells were treated with DMSO (control), BI-2536 (4 nmol l^−1^), fasudil (20 μmol l^−1^) or BI-2536/fasudil. Equal amounts of proteins from cell lysates were subjected to western blotting analyses. The numbers underneath the blotting bands represent the normalized density quantified by densitometry using ImageJ 2 × software. The relative mRNA levels of p21^WAF1/CIP1^ after normalization to β-actin expression were determined by quantitative PCR. The error bars correspond to the s.d.'s from three independent experiments. (**b**) The levels of p21 protein and mRNA expression in treated A549 and H522 cells. The error bars correspond to the s.d.'s from three independent experiments. (**c**) BI-2536/fasudil-mediated p21 activation was independent of p53 regulation. p21 and p53 were probed in isogenic HCT-116 (p53^+/+^) and HCT-116 (p53^−/−^) cells. (**d**) Immunoblot analysis of protein levels in the nucleus and cytoplasm in response to the indicated treatments in A549 cells. Histone H3 and α-tubulin served as nuclear and cytoplasmic fraction markers, respectively. (**e**) A549 cells were treated as indicated for 16 h. The nuclear accumulation of p21 was determined by immunofluorescence staining. The cells were stained with the anti-p21 antibody (green), nuclei were counterstained with 4,6-diamidino-2-phenylindole (blue) and F-actin was stained with phalloidin (red). Immunofluorescence was recorded using confocal laser fluorescence microscopy. Scale bars, 20 μm. (**f**) A549 cells were transfected with p21 siRNA or a control siRNA for 24 h, and then exposed to single drugs or to BI-2536/fasudil 48 h. The cell cycle distribution was analysed by flow cytometry using propidium iodide staining. The efficiency of p21 knockdown was examined by immunoblotting. (**g**) HCT-116 (*KRAS* mutant and p21 wild type) cells and its p21 knockout counterparts (HCT-116 p21^−/−^) were exposed to single drugs or to BI-2536/fasudil respectively. The cell cycle distribution was analysed. The efficiency of p21 knockout was examined by immunoblotting.

On the basis of the results of the microarray analysis, we further evaluated the effect of BI-2536/fasudil on the p53 signalling pathway. While this combination had little impact on p53 protein levels, it acutely increased the level of the cyclin-dependent kinase inhibitor p21^WAF1/CIP1^ in *KRAS*-mutant T29Kt1 cells, but not in the isogenic wild-type T29 cells (Fig. 4a). To further confirm the differences of p21 messenger RNA (mRNA) expression in *KRAS*-mutant and wild-type cells, we conducted quantitative PCR (qPCR) analyses in these cells. We found that treated *KRAS*-mutant cells had much higher levels of p21 compared to wild-type ones (Fig. 4a). Similar effects were observed in A549 (*KRAS*-mutant) and H522 (*KRAS* wild-type) cells; increased p21 protein and mRNA expression predominantly occurred in *KRAS*-mutant cells (Fig. 4b; Supplementary Fig. 2b).

Interestingly, we found that the level of the pro-apoptotic protein BAX was increased, while CDK1 phosphorylation (Tyr15) was decreased in *KRAS*-mutant cells in response to the BI-2536/fasudil combination (Supplementary Fig. 2c). These results agree with the model that p21 promotes G2/M cell cycle arrest and apoptosis through BAX and CDK1 (ref. 34), and are consistent with our earlier results (Fig. 2c,d). As expected, under the same conditions, p53 protein levels were not apparently affected in *KRAS*-mutant cells, and we therefore speculate that the BI-2536/fasudil-mediated upregulation of p21 is independent of p53.

To further verify our assumption, we used two *KRAS*-mutant human colon cancer cell lines: HCT-116 cells, which have wild-type p53 (p53^+/+^) and a subline that is deficient in p53 (HCT-116 p53^−/−^), to explore whether p21 activation was mediated by p53 in response to BI-2536/fasudil. Western blotting showed that p53 deficiency did not impair the BI-2536/fasudil-induced upregulation of p21 (Fig. 4c). These results suggested that p21 plays an independent role in the efficacy of this combined treatment in *KRAS*-mutant cells.

Because the growth inhibitory function of p21 is associated with its nuclear localization^34^, we further investigated the subcellular localization of p21 in response to drug treatments. We analysed its protein expression in the cytoplasm and in the nuclear extracts, and found that BI-2536/fasudil caused higher expression and nuclear translocation of p21 in *KRAS*-mutant A549 cells compared with either agent alone (Fig. 4d). Co-treatment with BI-2536 and fasudil resulted in a significant increase in p21 nuclear accumulation and a marked decrease in cytoplasmic p21 by immunofluorescence assays (Fig. 4e), which is consistent with the data shown in Fig. 4d. In addition, the p21 nuclear shift mediated by the drug combination was consistent with the observed survival inhibition in *KRAS*-mutant cells (Fig. 2b).

To confirm the significant role of p21 in the efficacy of BI-2536/fasudil, we silenced p21 expression by RNAi and the CRISPR/CAS9 system, and analysed its effects on the cell cycle progression in *KRAS*-mutant cancer cells. As expected, knockdown of p21 led to a significant attenuation of BI-2536/fasudil-induced G2/M arrest (Fig. 4f), while total depletion of p21 led to a complete rescue of G2/M arrest mediated by the drug pair in *KRAS*-mutant cancer cells (Fig. 4g). These findings unambiguously assign a critical role for p21 in the combinatorial action. Taken together, our results suggested that the growth inhibitory effect of BI-2536/fasudil in cells carrying a *KRAS* mutation was mediated by p53-independent p21 activation.

### The *in vivo* therapeutic efficacy of BI-2536/fasudil

On the basis of the broad efficacy of combined PLK1 and ROCK inhibition against *KRAS*-mutant cells *in vitro*, we assessed the therapeutic efficacy of this combination in a series of preclinical cancer models *in vivo*.

**Figure 5:**
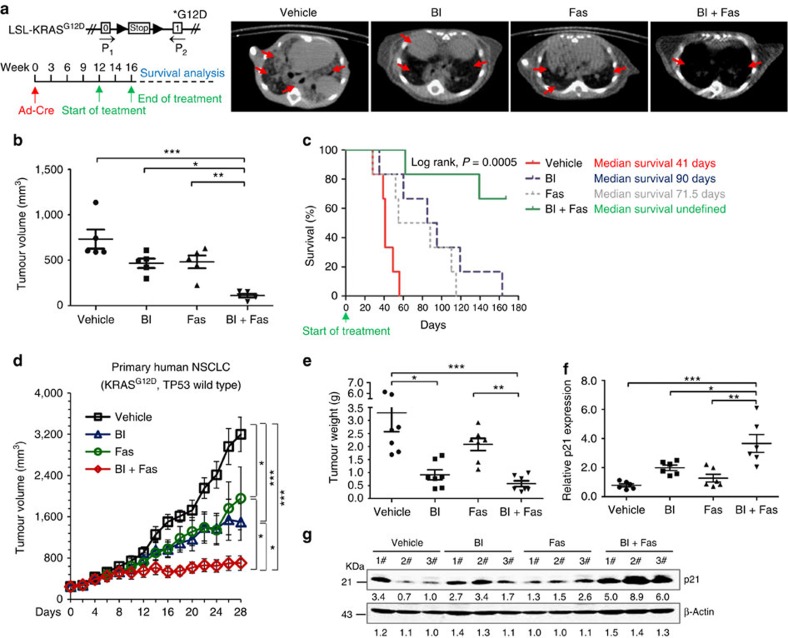
*In vivo* efficacy of combined PLK1 and ROCK inhibition. (**a**) Left: schematic illustration of the LSL-KRAS^G12D^ allele and drug treatment protocol. Established lung tumours in LSL-KRAS^G12D^ mice were treated with vehicle, BI-2536 (BI), fasudil (Fas) or both drugs in combination (BI+Fas). Right: after 4 weeks treatment, animals were scanned by micro-computed tomography and representative transverse images are shown. Red arrows indicate lung tumours. (**b**) Mean tumour volume at the end point of the indicated treatments. Each dot represents an individual mice (*n*=5 per group). Data represent the mean±s.e.m. *P<0.05; **P<0.01; ***P<0.001 by one-way analysis of variance (ANOVA) followed by Bonferroni multiple comparison test. (**c**) The survival rate was calculated by Kaplan–Meier method (*n*=6 per group). Statistical significance was assessed by log-rank tests, *P<0.05; **P<0.01; ***P<0.001. (**d**) The growth of primary NSCLC tumour xenografts (*n*=8 per group). Data represent the mean±s.e.m. *P<0.05; **P<0.01; ***P<0.001 by one-way ANOVA followed by Bonferroni multiple comparison test. (**e**) Tumour weights of the primary NSCLC tumour xenografts upon euthanasia at day 28. Each dot represents a tumour from an individual mouse. Data represent the mean±s.e.m. *P<0.05; **P<0.01; ***P<0.001 by one-way ANOVA followed by Bonferroni multiple comparison test. (**f**) p21 mRNA expression in the primary NSCLC tumour xenografts. Each dot represents a tumour from an individual mouse (*n*=6 per group). Data represent mean±s.e.m. *P<0.05; **P<0.01; ***P<0.001 by one-way ANOVA followed by Bonferroni multiple comparison test. (**g**) p21 protein level in the lysates from the tumours in the primary tumour xenograft mice. Each number represents a tumour from an individual mouse. See also Supplementary Fig. 3.

First, we assessed the efficacy of combined PLK1/ROCK inhibition in an acknowledged and widely used mouse model for KRAS study, the LSL-KRAS^G12D^ mouse model (Fig. 5a, upper left)^35^,^36^. After 12 weeks of cancer progression induced by Adenovirus-Cre, mice were randomly treated with vehicle, BI-2536, fasudil or combination of both the drugs for 4 weeks and then measured by micro-computed tomography (Fig. 5a, lower left). BI-2536/fasudil led to significantly greater tumour regression compared with vehicle or either agent alone, with near-complete abrogation of lung tumours in some cases (Fig. 5a, right; Fig. 5b). Infected LSL-KRAS^G12D^ mice began to die on day 28 with a median survival of 41 days. Therapies with single drug led to a modest survival advantage (median survival of 90 days in BI-2536 group; median survival of 71.5 days in fasudil group). Strikingly, the combined therapy led to much higher long-term survival advantage (log-rank test; *P*=0.0005; Fig. 5c).To facilitate the translation of this novel drug pair into clinical settings, we next tested the combination in a patient-derived tumour explant (PDTX) model of lung cancer carrying an activating G12D *KRAS* mutation. After 4-week treatment, the co-administration of BI-2536 and fasudil led to a marked tumour growth inhibition (*P*<0.0001, one-way analysis of variance followed by Bonferroni multiple comparison test) compared with the vehicle or either single agent alone, suggesting a strong synergy (Fig. 5d,e; Supplementary Fig. 3a). The effect of this combination therapy was quite durable, and all treatments were well tolerated because no animals in these studies exhibited significant differences in body weight between the groups (Supplementary Fig. 3b). Similar results were observed in the A549 orthotopic lung cancer model (Supplementary Fig. 3c–f). Overall, these results indicate that BI-2536/fasudil has substantial preclinical *in vivo* efficacy in different types of *KRAS*-mutant cancer models and provide a rationale for this combination in clinical trials.

To further evaluate the mechanism that underlies the anti-proliferative effects of this combination *in vivo*, PDTX tumour samples were collected for qPCR and immunoblotting analyses. Consistent with the results in tumour cells *in vitro*, the combination of BI-2536 and fasudil led to significant increases in the mRNA and protein expression of p21 in PDTX tumours (Fig. 5f,g). These findings supported the hypothesis that the efficacy of BI-2536/fasudil in *KRAS* tumour regression was mediated by p21 activation.

### The overexpression of p21^WAF1/CIP1^ impairs *KRAS*-mutant cancer

**Figure 6:**
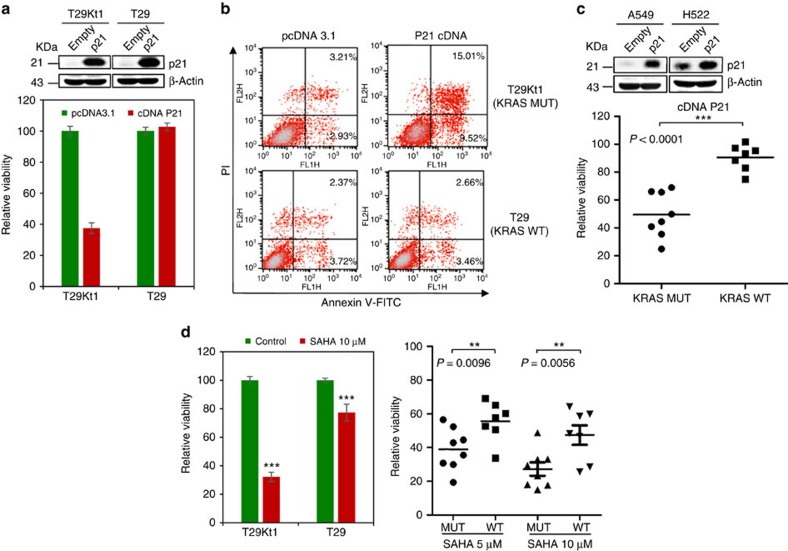
P21^WAF1/CIP1^ overexpression impairs the survival of *KRAS*-mutant cancer cells. (**a**) T29Kt1 and T29 cells were transfected with p21 cDNA plasmid or pcDNA 3.1 empty vector. Cell viability was measured 48 h after transfection. Data represent the mean±s.d. from three independent experiments. The efficiency of p21 overexpression was examined by immunoblotting. (**b**) T29Kt1 and T29 cells were transfected with p21 cDNA plasmid or pcDNA 3.1 empty vector. The percentage of apoptotic cells (Annexin positive) was determined by Annexin-V and propidium iodide staining. (**c**) Cell viability was measured in various cancer cells after p21 cDNA transfection. The dots represent the relative cell viability. The bars indicate the means. Student's *t*-tests were performed between the MUT and WT groups; **P<0.01; ***P<0.001. The efficiency of p21 overexpression in representative cells were examined by immunoblotting. (**d**) T29Kt1, T29 cells and different cancer cell lines were treated with SAHA or vehicle control for 48 h. Cell viability was measured. The columns indicate the means, and the bars indicate the s.d. The dots represent relative cell viability. Student's *t*-tests were performed between the MUT and WT groups; **P<0.01; ***P<0.001. MUT, mutant; WT, wild type.

Our results strongly indicated that tumour cells and xenografts with activated *KRAS* relied on suppressing p21 activity for growth. We hypothesized that there is a synthetic lethal interaction between p21 and KRAS, and that the elevated expression of p21 would suppress *KRAS*-mutant cancers. Initial support for this hypothesis came from an analysis of the effects of p21 overexpression on the survival of *KRAS*-mutant and wild-type cells. Intriguingly, our data showed that the overexpression of p21 by transfection with a p21 complementary DNA (cDNA) plasmid led to an apparent inhibition of cell viability in *KRAS*-mutant T29Kt1 cells but not in their *KRAS* wild-type counterparts (Fig. 6a). The overexpression of p21 induced marked cell apoptosis in *KRAS*-mutant T29Kt1 cells (Fig. 6b). We then extended these studies to a panel of cancer cell lines. Similar results were observed; cells harbouring *KRAS* mutations tended to be much more sensitive to p21 overexpression than cells with wild-type *KRAS* (Fig. 6c; *P*<0.0001, Student's *t*-tests).

Several anticancer agents exert their function, in part, through the induction of p21, such as histone deacetylase inhibitors and statins^34^,^37^. We further assessed the impact of SAHA, a histone deacetylase inhibitor, on *KRAS*-mutant cancer cells. SAHA selectively inhibited *KRAS*-mutant cells (Fig. 6d). In summary, our data supported the hypothesis that p21 activation could serve as a synthetic lethal factor in *KRAS*-mutant cancer cells and that p21 itself may be a novel therapeutic target for cancers with *KRAS* mutations.

## Discussion

Although *KRAS* is commonly mutated in human cancers, targeted therapies for these refractory cancers remain a major clinical challenge. Innovative approaches have recently been developed using small molecules to impair the interaction between KRAS and PDEδ or effectors^9^,^38^, but these molecules are a long way from use in the clinic. Mutant *KRAS* persistently and directly activates both RAF and PI3K, which has led to the exponential growth of studies aiming to treat *KRAS*-mutant tumours by coordinately inhibiting both of these pathways^39^. Unfortunately, the toxicity of this combination restricts its clinical applicability^8^,^23^. In recent years, synthetic lethal analyses have made another big splash and successfully uncovered multiple non-oncogene addiction pathways that were required for the survival of *RAS*-mutant cells^17^,^20^. Although the functions of most KRAS synthetic lethal genes have not been systematically validated, the potential utility of these genes is promising^18^. As a complementary approach, synthetic lethal chemical screening opens a new avenue for the therapeutic treatment of *KRAS*-transformed tumours. More importantly, it provides a shortcut and affords easy translational strategies utilizing drugs that are already in clinical use.

In this study, we conducted a synthetic lethal chemical screening and a combinatorial strategy to identify novel drug pairs that would effectively kill *KRAS*-mutant cancers. Our screening revealed 65 combination hits with a CI<1. Because a CI<0.7 depicts a strong synergy, this cluster of hits was further analysed. Although the number of agents in each RAS-related catalogue was relatively small in our screening program, several previously reported synergistic combinations for the treatment of *KRAS*-driven cancers were confirmed, such as the combined inhibition of MEK and PI3K^39^ (CI=0.59 at median effective dose), the combined inhibition of BCL-XL and MEK^23^ (CI=0.68 at median effective dose), the combined inhibition of Hsp90 and MEK^40^ (CI=0.03 at median effective dose) and the combined inhibition of MEK and AKT^41^ (CI=0.77 at median effective dose), which was recently set for a clinical trial (Trial NCT number NCT01021748). This synergistic cluster of CI<0.7 also included previously described combination strategies in cancer therapy regardless of *KRAS* status, such as the combination of topoisomerase inhibitors with tyrosine kinase inhibitors^42^,^43^ or mTOR inhibitors^44^ in different cancer models. Importantly, our screening strategy also identified previously undescribed drug interaction patterns and several novel combinations with high efficacy against *KRAS*-mutant cancers, for example, the combined inhibition of PLK1 and ROCK (CI=0.33 at median effective dose). We classified the gene types in the synergy hits (CI<1; Fig. 1a) and found that 71% of the combinations contained drugs whose targets were synthetic lethal genes of oncogenic *KRAS*, suggesting that *RAS*-transformed cells relied heavily on additional signalling contexts for survival. These findings are fully supported by the concept of non-oncogene addictions in cancer cells^5^, which provides the rationale for developing synthetic lethality-based anti-RAS therapy.

Given the importance of synthetic lethal genes in buffering the effect of changes in *KRAS*, combinations of inhibitors that target these genes may be promising strategies to treat recalcitrant *KRAS*-mutant cancers. In our combinatorial screening, combined PLK1/ROCK inhibition with BI-2536 and fasudil emerged as a top hit and exhibited a strong ability to induce apoptosis and cell cycle arrest in *KRAS*-mutant cancer cell lines. Importantly, this combination produced potent tumour regressions in a series of *KRAS*-mutant lung cancer models *in vivo*, such as LSL-KRAS^G12D^ mouse model and PDTX models, as well as orthotopic lung cancer models (Fig. 5; Supplementary Fig. 3). Specially, this drug pair markedly increased the survival rate of LSL-KRAS^G12D^ mice as compared with either single agent alone, suggesting an encouraging therapeutic window. BI-2536 and fasudil are already being used in clinical trials^17^,^20^, the single agents or dual agents at the doses tested in this study were well tolerated, and no gross toxicity was observed in the tumour-bearing mice. Our findings increase the likelihood that the coordinate inhibition of synthetic lethal genes of oncogenic *KRAS* may provide a novel therapeutic approach for treating these recalcitrant cancers. Further study of this type of combination (for example, the dual inhibition of PLK1 and ROCK) against *KRAS*-mutant cancers in clinical trials may be warranted.

Microarray profiling revealed that the p53 signalling pathway was implicated in the synergistic efficacy of BI-2536/fasudil. The expression of p21, a key target gene of p53, was examined in treated cells and primary tumour xenografts. Intriguingly, we found that BI-2536/fasudil significantly elevated the expression of p21 *in vitro* and *in vivo*, but p21 activation was independent of the TF p53. These data were not paradoxical because p21, a critical cell cycle regulator, can be induced by p53-dependent and -independent mechanisms. Consistent with this, TFBS enrichment analysis revealed that, in addition to p53, TFs such as CREB, AP2, p300, E2F and STAT1 were all involved in the mechanism of this drug pair (Fig. 3c). These TFs have been reported to induce *CDKN1A* transcription in a p53-independent manner^34^. Notably, this combination did not affect the phosphorylation of AKT and ERK (Supplementary Fig. 2c), further indicating that it probably targeted a synthetic lethal dependency of mutant KRAS, rather than RAS downstream effectors. p21 depletion rendered cells resistant to the combination treatment (Fig. 4f,g), indicating that p21 operates orthogonally to the oncogene RAS. Synthetic lethal interactions have been most commonly described for loss-of-function alleles, and they may also involve gain-of-function alleles^45^. The concept of ‘synthetic dosage lethality' was proposed to identify genetic interactions by the overexpression of a particular reference gene in the background of a target mutation^46^. On the basis of this concept, *CDKN1A* (p21) and *KRAS* are considered to be ‘synthetic lethal' because the overexpression of *CDKN1A* combined with a mutation in *KRAS* resulted in a lethal phenotype in our system. Previous genome-wide studies^17^,^20^ to uncover KRAS synthetic lethality did not identify *CDKN1A* as a synthetic lethal gene in *KRAS*-mutant cancer, primarily because those screens were based on the loss-of-function interference rather than gain-of-function phenotypes. Since p21 protein plays an important role in tumour suppression, targeting p21 or factors that regulate its activity is a promising therapeutic strategy^34^,^37^. In this study, we revealed for the first time that p21 is a novel synthetic lethal candidate of oncogene *KRAS*. Overexpression of p21 by cDNA or drugs selectively killed *KRAS*-mutant cells (Fig. 6), suggesting that p21 is a potential target for the treatment of KRAS-mutant cancer.

Our results are, to some extent, consistent with previous findings that *KRAS*-mutant cells showed a particular dependence on genes implicated in mitotic functions, such as PLK1 (ref. 17), components of the anaphase-promoting complex^17^, CDC2/CDK1 (ref. 47) and CDC6 (ref. 21). Mutations in oncogenes, such as *RAS*, have been suggested to contribute to chromosome instability and cause mitotic stress, and slight disturbances in cell cycle regulation may therefore have a serious impact on survival^5^,^48^. The CDK inhibitor p21 is implicated in cell mitotic regulation due to its ability to stabilize CDK–cyclin complexes and proliferating cell nuclear antigen^34^. In this study, the combination of BI-2536 and fasudil markedly induced p21 expression. As a result of p21 induction, mitotic stress was augmented in *KRAS*-mutant cancers, and this stress may cause the observed susceptibility to apoptosis in *KRAS*-mutant cancers.

In summary, we developed a combinatorial drug screening based on synthetic lethality to identify novel combination partners for the treatment of *KRAS*-driven cancers. Our study revealed a preclinical rationale for exploring a synthetic lethal regimen that combined the inhibition of PLK1 and Rho signalling to conquer *KRAS*-mutant cancer. The activity of this drug pair was mediated by activating p21, and *CDKN1A* (p21) was further identified as a new ‘synthetic lethal partner' of *KRAS.* Our new combination strategy defined by targeting the synthetic lethal partners of the oncogene *KRAS* may warrant further clinical investigation.

## Methods

### Cell lines and culture

The immortalized human ovarian surface epithelial cells T29 and their transformed counterparts T29Kt1 (with the introduction of KRAS^V12^ into the immortalized cells) were reported previously^28^ and were cultured in medium consisting of 1:1 MCDB105 medium and M199 medium (Sigma-Aldrich Co.) with 10% fetal bovine serum (HyClone) in the presence of 1% penicillin and streptomycin (HyClone). HCT-116 (p53^+/+^) and HCT-116 (p53^−/−^) were kindly provided by Dr Chuangui Wang (East China Normal University, Shanghai, China) and were maintained in DMEM with the same supplements. The culture medium of other cells used is listed in Supplementary Table 2. A549, H441, H358, H1299, H1975, PC9, H661, H522, HCT-15, LoVo, SW480, SW620, DLD-1, HT29, CaCo2, HCT-8, CCD-18Co, CCD841CoN, Panc-1, CFPAC-1 and BxPC-3 were obtained from the American Type Culture Collection (Manassas, VA, USA); Calu-1, Calu-3, WI-38, MRC-5, T84, LS174T, SW1116 and AsPC-1 were obtained from the Shanghai Cell Bank of the Chinese Academy of Sciences (Shanghai, China). *KRAS* status identification by sequencing and limited genotyping in cancer cells was shown in Supplementary Table 2. Most of these cells were grown at 37 °C under a humidified 95:5 (%; v/v) mixture of air and CO_2_. SW480 and SW620 cells were grown without CO_2_. All of the cell lines were authenticated by short tandem repeat analysis.

### Cell viability assays

Cells were plated at a density of 2,000–5,000 cells per well in 96-well plates and allowed to adhere overnight, and subjected to drug treatment for 72 h. Cell viability was determined using CellTiter 96 AQ_ueous_ One Solution from Promega according to the manufacturer's instructions. The half inhibitory concentration IC_50_ values were calculated by Prism 5 (GraphPad Software). The drug combinations were performed with fixed drug ratios (that is, the IC_50_ ratio), and the CI equation as described by Chou-Talalay^49^,^50^ was generated using CalcuSyn software (Version 2; Biosoft). Combinations with CI_s_<1 were considered to be synergistic. The following small inhibitors were used (Supplementary Table 1): sorafenib (Cat. #S7397), selumetinib (AZD6244, Cat. #S1008), perifosine (Cat. #S1037), rapamycin (Cat. #S1039), NVP-BEZ235 (Cat. #S1009), gefitinib (Cat. #S1025), axitinib (Cat. #S1005), sunitinib (Cat. #S7781), PD0332991 (Cat. #S1116), BI-2536 (Cat. #S1109), BX-795 (Cat. #S1274), BAY 11–7082 (Cat. #S2913), bortezomib (Cat. #S1013), fasudil (Cat. #S1573), ABT-263 (Cat. #S1001), BI-6727 (Cat. #S2235), Y-27632 (Cat.#S1049) and SAHA (Cat. #S1047) were obtained from Selleck Chemicals. Irinotecan (Cat. #I1406) and topotecan (Cat.#T2705) were obtained from Sigma. Wortmannin was obtained from Calbiochem/Merck (#681676). 17AAG was obtained from Invivogen (#ant-agl-5). 3-BrPA was obtained from TCI (#M2223).

### Apoptosis

Cells were seeded in six-well plates and treated with the indicated treatments for 72 h. The apoptosis assays were conducted using the BD Pharmingen Apoptosis Detection Kit (BD) and analysed by flow cytometry (FACSCalibur, BD).

### Cell cycle analysis

Exponentially growing cells were incubated with 80–100 ng ml^−1^ of nocodazole (Sigma) for 16 h to synchronize the cell cycle. The synchronized cells were exposed to the indicated treatments for 48 h. The cells were then fixed with 70% ethanol overnight, permeabilized with 0.1% Triton X-100, and incubated with Ribonuclease A (RNase A; Takara biotechnology Co.) and propidium iodide (Sigma). The cell cycle was analysed by flow cytometry (FACSCalibur, BD).

### Two-dimensional clonogenic assay

A549 cells were plated at a density of 1,000 cells per well in six-well plates. After cell attachment, medium containing treatment was added and changed every 3 days for a period of 1 week. The colonies were fixed in 4% paraformaldehyde and stained with 0.5% crystal violet. Photographs were taken, and clones were counted if they contained >50 viable cells.

### RNA isolation and microarray analysis

A549 cells were treated with BI-2536 (4 nM), fasudil (20 μM) or the combination of BI-2536 and fasudil for 6 h. Samples were obtained from three independent biological replicates. Total RNA was isolated using RNAiso plus (TaKaRa) according to the manufacture's protocol. A total of 10 μg purified RNA from each sample was submitted to the Gene Tech Company Limited (Shanghai, China) for labelling and hybridization using Affymetrix GeneChip PrimeView Human Gene Expression array (Affymetrix). The microarray data discussed in this article have been deposited in National Center for Biotechnology Information Gene Expression Omnibus (GEO) and are accessible through (GEO) Series accession number GSE60887 (http://www.ncbi.nlm.nih.gov/geo/query/acc.cgi?acc=GSE60887).

### Microarray data analysis

The robust multi-array average algorithm^51^ implemented in the Affymetrix Expression Console was applied on the raw intensity data to perform the inter-chip normalization. The moderated *t*-test^52^ in the limma package was then used to select the DEGs. The raw *P* values of results were corrected for multiple testing by BH method^53^.

To predict the activated TFs under the experiment condition, the TFBS enrichment analysis was performed as follow: let *k* denote the number of DEGs in *p* expressed genes. We count the total number *X* of TFBS for the TF *a* from all the promoter regions of the *k* DEGs. We randomly picked up *k* genes from *p* expressed genes and counted the number *x* of TFBS for the same TF *a*, this procedure was repeated *n* times. The significance of the TFBS enrichment analysis for the TF was defined as:


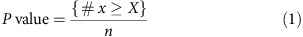


The TFBS data from the UCSC TFBS conserved track were used in this study and *n* was set to 10^5^ to accurately estimate the *P* value. The promoter region was defined as the −5- to 1-kb region relative to the transcription start site.

### Western blotting and antibodies

Western blotting was performed using standard methods. After being treated with indicated drugs, whole-cell or tumour tissue extracts were prepared by radioimmunoprecipitation assay buffer (150 mM sodium chloride, 50 mM Tris pH 8.0, 1% Triton X-100, 0.5% sodium deoxycholate, 0.1% SDS) supplemented with 10 mM NaF, 1 mM Na_3_VO_4_, 5 mM EDTA, 1 mM EGTA, 5 μg ml^−1^ leupeptin, 1 μg ml^−1^ pepstatin A, 1 mM phenylmethylsulfonyl fluoride, and protease and phosphatase inhibitor cocktail (Calbiochem). The cytoplasmic and nuclear extracts were prepared as previously described based on the different concentration of NaCl (ref. 54). Lysates were centrifuged at 12,000 r.p.m. for 20 min at 4 °C. Protein concentration of the supernatants was determined using a BCA protein Assay Kit (Thermo Scientific). Equal amounts (30–50 μg) of the proteins were resolved by 8–12% SDS–polyacrylamide gel electrophoresis gels and transferred to nitrocellulose membranes (Millipore). Membranes were blocked in 5% bovine serum albumin for 1 h at room temperature, and then incubated with specific antibodies for different western blot analyses at 4 °C overnight. The bound primary antibodies were detected by secondary conjugates compatible with infrared detection at 700 and 800 nm, and membranes were scanned using the Odyssey Infrared Imaging System (Odyssey, LI-COR). Representative blots are shown from several experiments. The relative optical density of blotting bands was quantified by Image J software (NIH, Bethesda, MD, USA) and normalized to the control.

The following antibodies were used in these assays, all diluted in Primary Antibody Dilution Buffer (Beyotime Biotechnology,China): antibodies directed against p53 (#9282), Pan-ERK (#9102), Pan-AKT (#9272), Histone H3 (#4499, 1:2,000 dilution), α-tubulin (#2125), Phospho-CDK1 (at Tyr15 site, #4539), Phospho-AKT (at Ser473 site, #9271) and Phospho-ERK (at Thr202/Tyr204 sites,#4376) were obtained from Cell Signaling Technologies (1:1,000 dilution). Antibodies directed against p21 (ab109520, 1:2,000 dilution) and BAX (ab32503, 1: 2,000 dilution) were obtained from Abcam. Anti-β-actin antibody was purchased from Sigma (A5441, 1:10,000 dilution). Secondary antibodies were from LI-COR Biosciences, including IRDye 800CW Goat anti-Mouse IgG (926–32210, 1:10,000 dilution) and IRDye 800CW Goat anti-Rabbit IgG (926–32211, 1:10,000 dilution). Uncropped blots are shown in Supplementary Fig. 4.

### RT–PCR and quantitative real-time PCR

The total RNA was isolated with RNAiso plus (TaKaRa). One microgram of total RNA was used for the synthesis of first-strand cDNA using the PrimeScript RT reagent kit (TaKaRa). Reverse transcription–PCR (RT–PCR) was performed in a 20-μl reaction mixture containing cDNA (1 μl), forward and reverse primers (200 nM each), dNTP (250 μM), 10 × PCR buffer (2 μl) and 0.2 μl of TaKaRa Taq (TaKaRa). The amplified DNA fragments were electrophoresed on an agarose gel. Quantitative real-time PCR was performed according to the manufacturer's instructions of the SYBR Premix Ex Taq kit (TaKaRa) on an Mx3005P thermal cycler (Stratagene). The forward and reverse primers for CDKN1A (p21) were as follows: forward, 5′-TGTCCGTCAGAACCCATGC-3′ and reverse, 5′ -AAAGTCGAAGTTCCATCGCTC-3′; β-actin: forward, 5′-AGCCGTGTTCTTTGCACTTT-3′ and reverse, 5′-AGGAAGGAAGGCTGGAAGAG-3′.

### siRNA experiments

A549 cells were plated in six-well plates and allowed to adhere for 24 h. For transfection, 6 μl of Lipofectamine TM 2000 reagent (Invitrogen) was added to 80 nM p21 small interfering RNA (siRNA) or negative control siRNA in a final volume of 1 ml culture medium. After 36 h of transfection, cells were either treated with the appropriate drug for cell cycle assay or lysed for immunoblotting to determine the efficiency of the knockdown. The siRNA for p21 and the negative control siRNA were synthesized and purified by GenePharma (Shanghai, China). The two siRNA sequences for human p21 used in this study were 5′-GAUGGAACUUCGACUUUGUUU-3′ (p21 siRNA-1) and 5′-GGACACAAAACCAGAGUUA-3′ (p21 siRNA-2).

### Construction of p21 knockout cell lines

The SpCas9 targeting vector lentiCRISPR v2 was purchased from Addgene (Cambridge, MA). For sgRNA cloning, the lentiCRISPR v2 vector was digested with BsmBI and ligated with BsmBI-compatible annealed oligos. SgRNA (5′-CCGCGACTGTGATGCGCTAA-3′) was designed using Optimized CRISPR Design. Lentiviruses were produced by co-transfection of 293T cells with lentiviral backbone constructs (lentiCRISPR v2) and packaging vectors (pMD2.G and pSPAX2) using Lipofectamine 2000 following to the manufacturer's protocols. Supernatant was collected 48 h post transfection and added to HCT-116 cells in a six-well plate. Infected cells were selected with puromycin, and single-cell clones with frame-shift mutation of P21 were chosen.

### Transfection of p21 expression plasmid

pcDNA 3.1 expression vectors encoding human p21^WAF1/CIP1^ were obtained from Dr Xiaotao Li (East China Normal University, Shanghai, China). This plasmid, along with an empty vector control were transfected into a panel of cell lines using Lipofectamine TM 2000 reagent (Invitrogen) according to the manufacturer's instructions. After 48 h of transfection, cell viability was measured using CellTiter 96 AQ_ueous_ One Solution from Promega (Madison, WI) as described above. Transfected cells were either conducted for RT–PCR assay to determine the efficiency of the overexpression. Cell lines used in this section were as follows: T29Kt1, A549, H441, Calu-1, HCT-116, LS174T, HCT-15, SW480 and SW680 cells carrying *KRAS* mutation; and T29, H522, H661, H1975, CaCO2, HT29, HCT-8 and BXPC-3 cells harbouring wild-type *KRAS*.

### Immunofluorescence assay

The nuclear translocation of p21 was examined by immunofluorescence. A549 cells were plated on glass coverslips and treated with indicated drugs. The cells were then fixed with 4% paraformaldehyde and permeabilized with 0.2% Triton X-100. The cells were washed in PBS, blocked and incubated with p21 antibody (1:200 dilution) at 4 °C overnight. After that the cells were incubated with goat anti-rabbit IgG-FITC secondary antibody for 1 h, stained with rhodamine–phalloidin (Molecular Probes) for 1 h and then counterstained for the nuclei with DAPI (50 ng ml^−1^) for 5 min. The images were acquired using a laser confocal microscope (Leica TCS SP5).

### Animal studies

C57BL/6 and BALB/cA nude mice used in the present study were purchased from National Rodent Laboratory Animal Resources (Shanghai, China). Animals were caged in the groups of five in a laminar airflow cabinet under specific pathogen-free conditions, fed with sterilized food and water, and kept on a 12-h light/dark cycle. All treatments were administered according to the guidelines of Institution Animal Care and Use Committee and all the protocols were approved by East China Normal University.

LSL-KRAS^G12D^ mice in a C57BL/6 background were purchased from the Jackson Laboratory (#008179; Sacramento, CA). These mice were crossed with wild-type C57BL/6 mice to yield sufficient genotype verified LSL-KRAS^G12D^ mice. To activate Kras^G12D^ in mouse lung, intranasal administration of Ad-Cre was performed as described^35^,^36^. Briefly, the verified 6-week-old male LSL-KRAS^G12D^ mice were anaesthetized with isoflurane via a gas chamber. Ad-Cre (purchased from HanBio, Shanghai, China) at the dose of 5 × 10^7^ plague-forming unit in a total volume of 125 μl and add 2 M CaCl_2_ to obtain a final concentration of 10 mM as coprecipitates. Ad-Cre nasally is delivered by using two 62.5-μl instillations with a 5-min interval. BI-2536 was dissolved in 0.1 N HCl per saline at 20 mg kg^−1^ and delivered intravenously under a twice per week schedule. Fasudil was dissolved in sterile water and delivered daily by oral gavage at a dosage of 50 mg kg^−1^. Mice were treated with indicated drugs or vehicles for 4 weeks and then measured by micro-computed tomography (PerkinElmer, Waltham, MA). The survival rate was calculated by Kaplan–Meier method. Statistical significance was assessed by log-rank tests.

The primary *KRAS*-mutant lung cancer mouse model was established as previously described^55^. Surgical samples of patient primary NSCLC presenting adenocarcinoma histology were obtained from treatment-naive patients at the Shanghai Changzheng Hospital (Shanghai, China). Prior written informed consent was obtained from all patients, and the study protocol was approved by the Shanghai Changzheng Hospital (Shanghai, China) ethics committee. In brief, surgically removed tumour tissues were cut into fragments of ∼15 mm^3^ and implanted using a trocar needle subcutaneously into two to three male NOD/SCID mice (5–6-week old) within 2 h. Transplanted mice bearing xenografts were observed weekly. When tumour volume reached ∼1,000 mm^3^, primary xenografts at the exponential growth phase were removed by serially passage to other 5–6-week-old male BALB/cA nude mice. After three consecutive mouse-to-mouse passages, the xenograft was considered to be stabilized and submitted to mutation analyses to verify that it consistently maintained clinical NSCLC molecular features. ‘Hot spot' mutations in EGFR (exons 18, 19 and 21), KRAS (exons 2 and 3), PIK3CA (exons 9 and 20) and BRAF (exon 15) were screened by direct sequencing. Numerous samples from early passages were immediately stored in liquid nitrogen and used for further experiments. Xenografts at passage 3 were used in this study. Tumours were grown until up to 250 mm^3^ before treatments. Mice were treated using the same conditions as in the LSL-KRAS^G12D^ mouse model. Tumour size was measured by calipers every other day and the body weight was measured in parallel. Volumes were determined by the formula: volume=length × width^2^ × 0.52. On day 28, 2 h following the final dosing, the mice were killed and tumour tissue was excised, weighed and snap-frozen in liquid nitrogen for western blotting and qPCR analysis.

### Statistical analysis

The data are presented as the mean±s.d. unless otherwise stated. Statistical tests were performed using Microsoft Excel and GraphPad Prism Software version 5.0. For comparisons of two groups, a two-tailed unpaired *t*-test was used. For comparisons of multiple groups, one-way analysis of variance was used. A Log-rank test was performed for survival curves. The levels of significance were set at *P*<0.05 (*), *P*<0.01 (**) and *P*<0.001 (***). The specific tests applied are included in the figure legends.

## Additional information

**How to cite this article:** Wang, J. *et al*. Suppression of KRas-mutant cancer through the combined inhibition of KRAS with PLK1 and ROCK. *Nat. Commun.* 7:11363 doi: 10.1038/ncomms11363 (2016).

## Supplementary Material

Supplementary Information Supplementary Figures 1-4, Supplementary Tables 1-3 and Supplementary Methods.
