# Supplementary material for: Suppression of KRas-mutant cancer through the combined inhibition of KRAS with PLK1 and ROCK
Source: Nat Commun. 2016 May 19;7:11363. doi: 10.1038/ncomms11363 (PMC4873974; doi:10.1038/ncomms11363)
Supplement: Supplementary Information — Supplementary Figures 1-4, Supplementary Tables 1-3 and Supplementary Methods. [file ncomms11363-s1.pdf]

Supplementary Figures

Supplementary Figure 1

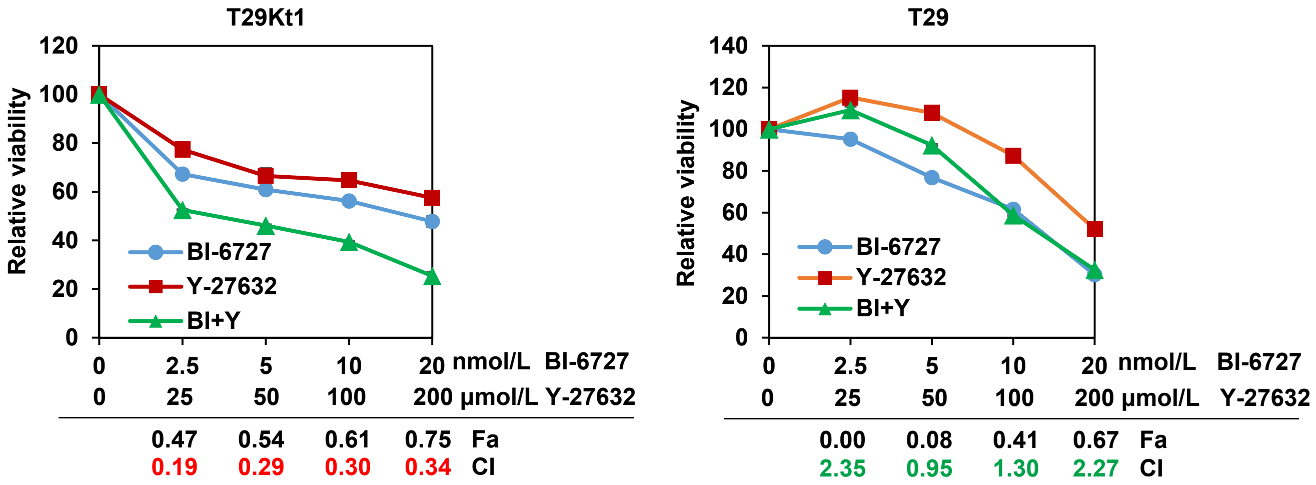

Supplementary Figure 1. The combined cytotoxicity of BI-6727 and Y-27632. T29Kt1 and T29 cells were incubated with increasing concentrations of BI-6727 (a PLK1 inhibitor) and Y-27632 (a ROCK inhibitor) alone or in combination for 72 hours, and the cell viability was determined. The CI and Fa values for the combination of BI-6727 and Y-27632 were calculated by using CalcuSyn software (Version 2; Biosoft). The averages and error bars represent the mean ± S.D from three independent experiments.

Supplementary Figure 2

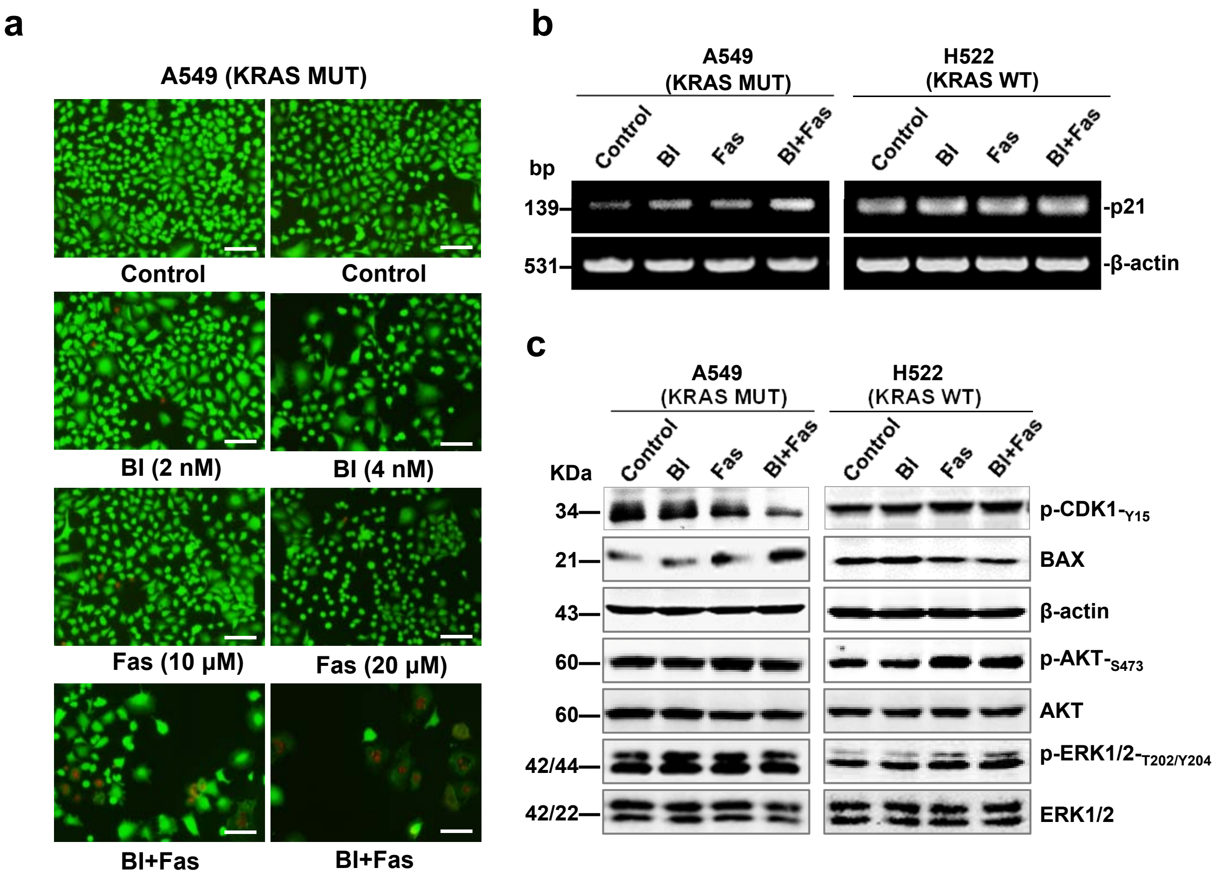

Supplementary Figure 2. Pharmacologic inhibition of PLK1 and ROCK in cancer cell lines. (a) Survival of A549 cancer cells was determined by Calcein-AM (green, viable cells) and Ethidium homodimer III (red, non-viable cells) double staining after treatment (Related to Figure 2b). Representative fluorescence images were shown. Scale bars, 200 μm. (b) A549 and H522 cells were treated with indicated drugs for 6 hours. p21WAF1/CIP1 mRNA expression was examined by RT-PCR (Related to Figure 4). (c) A549 and H522 cells were treated with indicated drugs for 16 hours. Protein from the whole-cell lysates was subjected to immunoblotting assay (Related to Figure 4).

Supplementary Figure 3

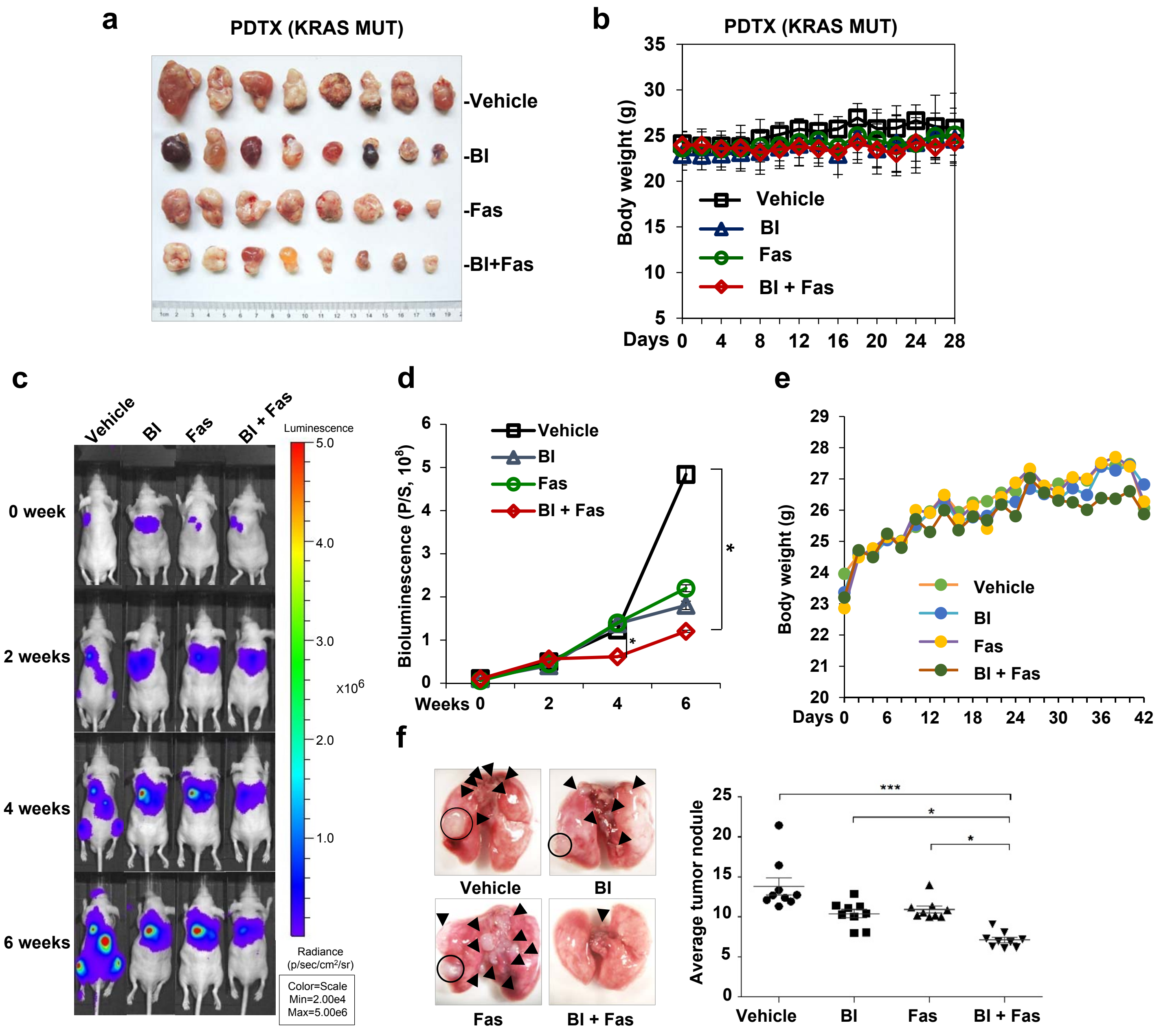

Supplementary Figure 3. ***In vivo*** **efficacy of combined PLK1 and ROCK inhibition, related to Figure 5.** (**a**) Gross images of solid tumors in patient derived tumor xenograft (PDTX) model. (**b**) Mouse body weight in PDTX model. (**c**) The progression of A545-luc orthotopic lung tumors (n = 9 mice per group) in BALB/c Nude mice. A representative bioluminescent image from each group was shown. (**d**) Quantitative bioluminescence analysis in mice bearing orthotopic lung tumors; P/S = photons/second; the error bars represent the SEM. (**e**) Mouse body weight in orthotopic lung tumor mouse model. (**f**) Average tumor nodule. Tumor nodules of 2–10 mm^3 in volume were counted using harvested lungs from the control and treated groups, and the average number of tumor nodules was determined. Each dot represents a tumor from an individual mouse. The tumor nodules in the lungs were indicated by arrows. *, $P < 0.05$; **, $P < 0.01$; ***, $P < 0.001$ by one-way ANOVA followed by Bonferroni multiple comparison test.

Supplementary Figure 4

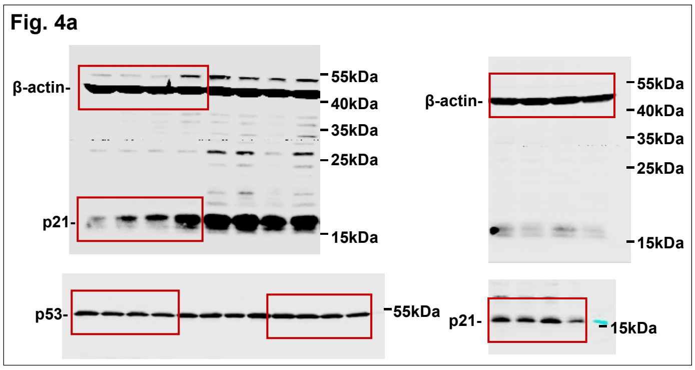

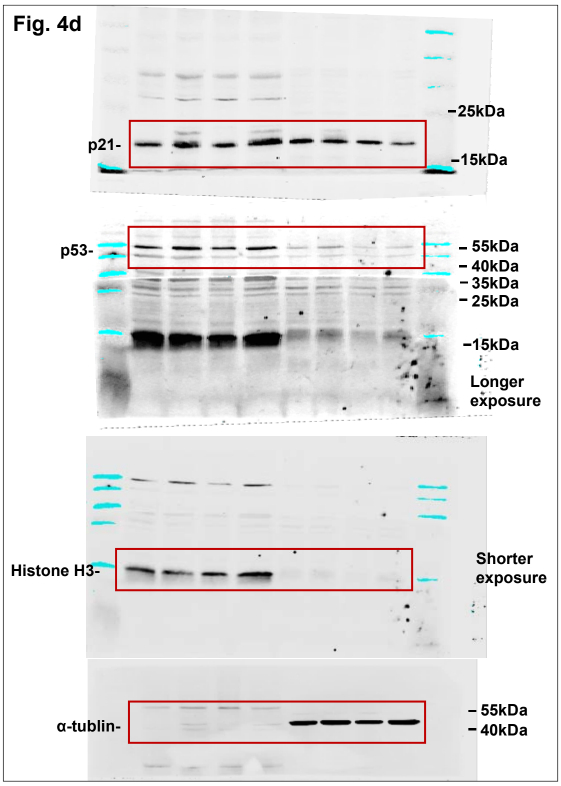

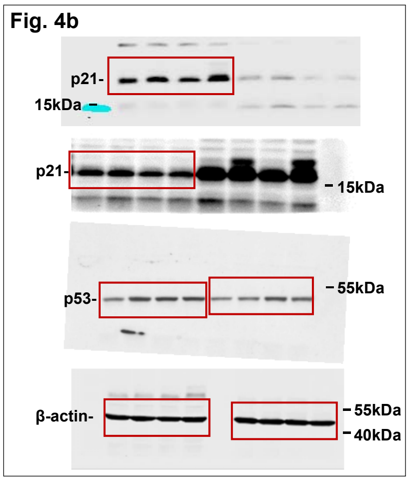

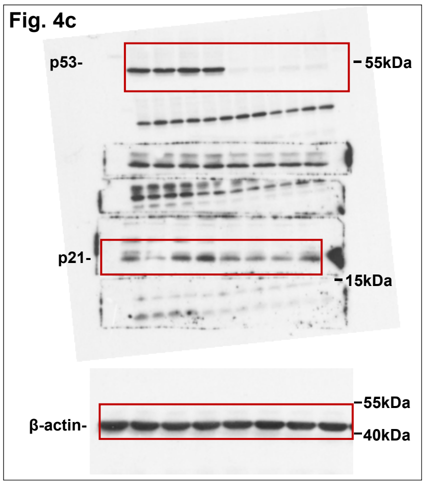

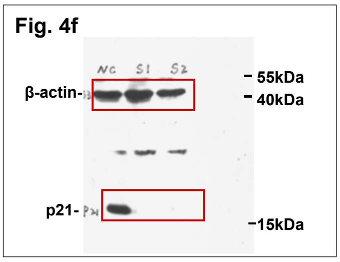

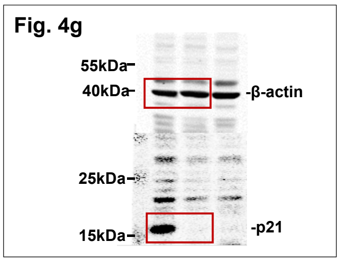

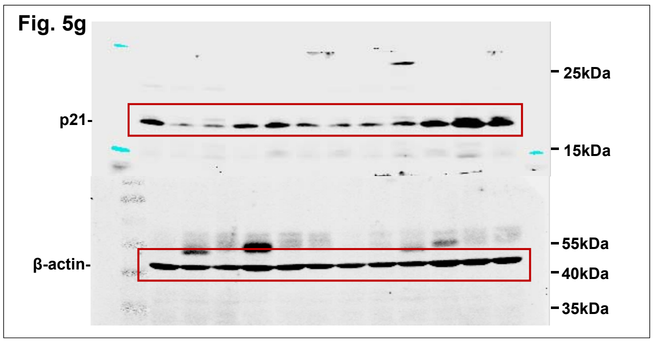

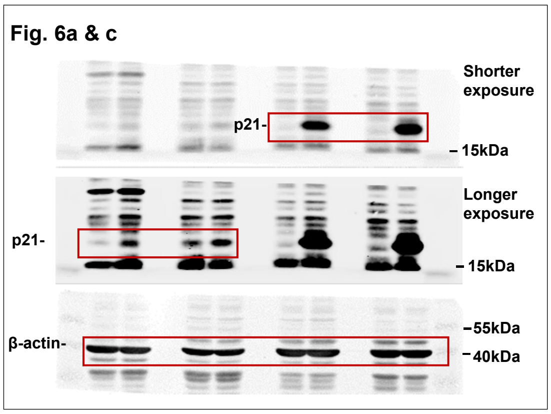

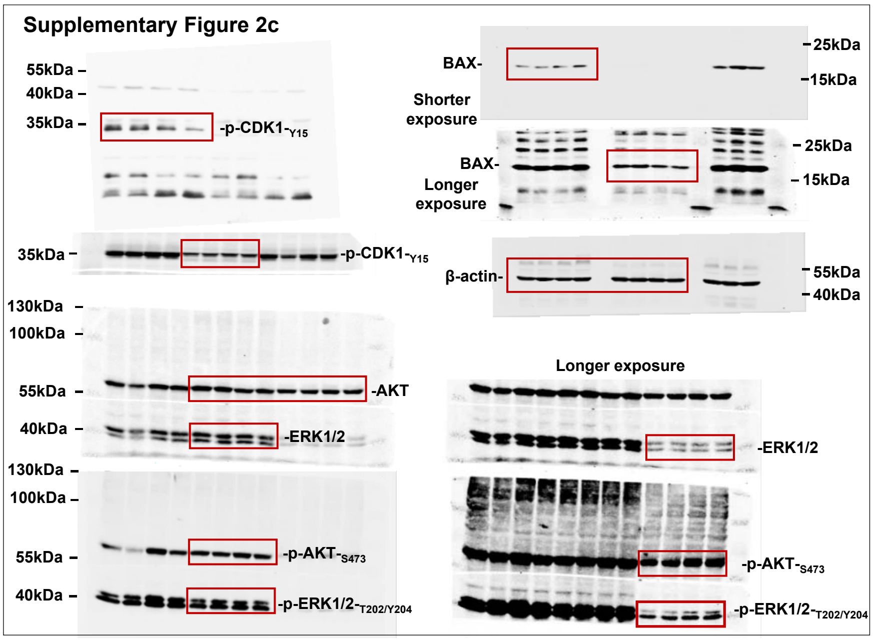

Supplementary Figure 4. Western blots for Figure 4, 5, 6 and Supplementary Figure 2.

Supplementary Tables

Supplementary Table 1. Drugs used in the screen and their primary targets (Related to Figure 1).

	**Drug**	**Target**	**Dose Range (μmol/L)**	**IC_{50}**	
				**T29Kt1**	**T29**
**RAF/MEK/ERK**	Sorafenib	RAF	1-20	7.17	14.28
	AZD-6244	MEK	10-200	153.30	NA
**PI3K/AKT/mTOR**	Wortmannin	PI3K	5-100	49.04	67.07
	Perifosine	AKT	1-40	27.37	NA
	Rapamycin	mTOR	1-40	19.47	39.41
	NVP-BEZ235	PI3K/mTOR	0.001-1	0.016	0.035
**RTK**	Gefitinib	EGFR	1-40	21.40	25.63
	Axitinib	VEGFR	1-40	9.70	NA
	Sunitinib	RTKs	1-20	6.499	19.06
**Synthetic Lethality**	PD0332991	CDK4	1-40	10.59	25.70
	BI-2536	PLK1	0.001-1	0.036	0.41
	BX-795	TBK1	0.5-10	10.65	8.458
	BAY11-7082	NFκB	1-20	4.716	4.836
	Bortezomib	Proteasome	0.001-0.008	0.0025	0.0018
	Irinotecan	Topoisomerase	1-40	26.60	48.47
	Topotecan	Topoisomerase	0.01-1	0.133	0.088
	Fasudil	ROCK	40-200	116.7	150.4
	ABT-263	BCL-XL	1-20	13.15	18.53
	17AAG	HSP90	0.1-2	0.8656	0.3859
	3-BrPA	Hexokinase 2	10-80	36.03	35.49

NOTE: Cell viability was measured. Concentrations required to inhibit 50% of cell growth (IC_{50}) were shown. Only primary drug targets are indicated. Abbreviation: NA, not assessed; $IC_{50,}$ concentrations required to inhibit 50% of cell growth.

Supplementary Table 2. Mutational profile and culture medium of cell lines.

Tissue	Cell Line	*KRAS* Status	*EGFR* Status	*TP53* Status	Medium
**Lung**	A549	p.G12S	WT	WT	DMEM
	H441	p.G12V	WT	MUT	RPMI-1640
	H358	p.G12C	WT	WT	RPMI-1640
	Calu-1	p.G12C	WT	WT	McCoy's 5a
	H1299	NRAS p.Q61K	WT	WT	RPMI-1640
	H1975	WT	p.L858R p.T790M	MUT	RPMI-1640
	PC9	WT	Exon 19 del	MUT	RPMI-1640
	H661	WT	WT	MUT	RPMI-1640
	H522	WT	WT	MUT	RPMI-1640
	Calu-3	WT	WT	MUT	EMEM
Normal	WI-38				EMEM
	MRC-5				EMEM
**Colon**	HCT116	p.G13D	WT	WT	DMEM
	HCT15	p.G13D	WT	MUT	RPMI-1640
	LoVo	p.G13D	WT	WT	F12-K
	SW480	p.G12V	WT	MUT	L-15
	SW620	p.G12V	WT	MUT	L-15
	DLD-1	p.G13D	WT	MUT	RPMI-1640
	T84	p.G13D	WT	MUT	DMEM/F12
	LS174T	p.G13D	WT	WT	DMEM
	SW1116	p.G12A	p.V654M	MUT	RPMI-1640
	HT29	WT	WT	MUT	McCoy's 5a
	CaCo2	WT	WT	MUT	EMEM
	HCT-8	WT		WT	RPMI-1640
Normal	CCD-18Co				EMEM
	CCD841CoN				EMEM
**Pancreas**	Panc-1	p.G12D	WT	WT	DMEM
	CFPAC-1	p.G12V	WT	MUT	IMDM
	AsPC-1	p.G12D	WT	MUT	RPMI-1640
	BxPC-3	WT	WT	MUT	RPMI-1640

NOTE: Cell lines and tumor type are shown. Specific mutations in *KRAS* and *EGFR* are displayed for each cell line. The status of *TP53* for each cell line is indicated as wild-type (WT) or mutated (MUT).

Supplementary Table 3. Enriched Transcription Factor Binding Sites (TFBS) of the differentially expressed genes (Related to Figure 3).

TF Name	p-value	q-value	GeneSymbo (s)	Family
CREB	0.00074	0.01328	CREB1	bZIP
AhR	0.00081	0.01328	AHR	bHLH
E4BP4	0.00096	0.01431	NFIL3	bZIP
MAZR	0.00119	0.01552	PATZ1	ZBTB
deltaCREB	0.00123	0.01552	CREB1	bZIP
AP-2gamma	0.00443	0.04100	TFAP2C	AP-2
p300	0.00444	0.04100	EP300	
p53	0.00459	0.04100	TP53	TP53
CHOP-10	0.00478	0.04100	DDIT3	bZIP
SREBP-1a	0.00506	0.04100	SREBF1	bHLH
SREBP-1b	0.00508	0.04100	SREBF1	bHLH
SREBP-1c	0.00517	0.04100	SREBF1	bHLH
E2F	0.00525	0.04100	E2F1,E2F2, E2F3, E2F4,E2F5, E2F6, E2F7, E2F8	E2F
STAT6	0.00649	0.04612	STAT6	STAT
c-Myb	0.00654	0.04612	MYB	MYB
STAT2	0.00675	0.04612	STAT2	STAT
Max1	0.01041	0.06829	MAX	bHLH
ITF-2	0.01246	0.07755	TCF4	bHLH
COUP-TF1	0.01283	0.07755	NR2F1	COUP/EAR
Hlf	0.01324	0.07755	HLF	bZIP
HNF-4alpha2	0.01407	0.07957	HNF4A	Thyroid hormone receptor
AP-2alphaA	0.01659	0.08754	TFAP2A	AP-2
STAT1	0.01666	0.08754	STAT1	STAT
c-Rel	0.01708	0.08754	REL	RHD
MIF-1	0.01807	0.08980	HIVEP2	zf-C2H2
SRY	0.01921	0.09266	SRY	HMG
E2F-1	0.02084	0.09765	E2F1	E2F
HSF1 (long)	0.02162	0.09849	HSF1	HSF
NF-1	0.02956	0.12376	NFIC	NF-I
RFX1	0.02960	0.12376	RFX1	RFX
E2F-2	0.03179	0.12376	E2F2	E2F
E2F-4	0.03205	0.12376	E2F4	E2F
FOXD3	0.03230	0.12376	FOXD3	Fork head
E2F-3a	0.03237	0.12376	E2F3	E2F
E2F-5	0.03245	0.12376	E2F5	E2F

POU2F1	0.03377	0.12587	POU2F1	POU
FOXO4	0.03838	0.13987	FOXO4	Fork head
Egr-1	0.04509	0.16076	EGR1	zf-C 2H2
STAT4	0.04987	0.17401	STAT4	STAT

Supplementary Methods

Live/dead assay. Cells survival was also determined by live/dead assay (Invitrogen Carlsbad, CA) by measuring intracellular esterase activity and plasma membrane integrity. A549 cells (5×10^4 cells) were cultured in 6-well plates and treated as indicated for 72h. For measurement of cellular viability, 2 μmol/L calcein-AM, a green fluorescent indicator of the intracellular esterase activity of cells, and 4 μmol/L EthD-1, a red fluorescent indicator of membrane-damaged (dead) cells, were added to each well, and the plates were incubated for 5 min in 5% CO2 at 37°C. Live cells (green) and dead cells (red) were detected by fluorescence microscopy at 100 × magnification. Representative fluorescence images were shown.

Orthotopic model of human NSCLC. Six- to eight-week old male BALB/cA nude mice were intraperitoneally (i.p.) injected with pentobarbital sodium (50 mg/kg) and fixed in the left lateral decubitus position after anesthesia. A 5-mm skin incision overlying the left chest wall was made and the left lung was visualized through the pleura. A total volume of 100 μL of A549-Luc cells (1×10^6) mixed with 1:1 growth factor-reduced Matrigel (BD Biosciences, San Jose, CA, USA) was injected into the upper margin of the sixth intercostal rib on the left anterior axillary line to a depth about 5 mm. After tumor cell injection, the wound was stapled and the mice were placed in the left lateral decubitus position and observed until complete recovery.

A week after the implantation of A549-luc cells, mice were divided into four groups (8-10 mice per group) based on initial bioluminescent imaging, and those mice received treatments on the same schedule as mentioned above for successive 6 weeks. The concentrations of BI-2536 and fasudil administrated in this model were 20 mg/kg and 50 mg/kg, respectively.

Lung tumor development was monitored weekly by bioluminescent imaging using a Xenogen IVIS-200 Optical *in vivo* imaging system (Xenogen, Alameda, CA, USA). Ten minutes before acquiring *in vivo* images, animals

were i.p. injected with 150 mg/kg D-luciferin (Xenogen) diluted in PBS. All imaging procedures were performed under inhalation anesthesia using a mixture of 2.5% isoflurane/oxygen. Quantification of bioluminescence images was performed by determining ‘regions of interest’ (ROI) and the data were expressed as photon-flux (photons/sec/cm^2 /steradian). At the end of the experiment, animals were killed and lung tissues were resected and weighed.
